# Implementation and scale-up of a single-visit, screen-and-treat approach with thermal ablation for sustainable cervical cancer prevention services: a protocol for a stepped-wedge cluster randomized trial in Kenya

**DOI:** 10.1186/s13012-023-01282-3

**Published:** 2023-06-26

**Authors:** Michelle B. Shin, Lynda Myra Oluoch, Ruanne V. Barnabas, Colin Baynes, Harriet Fridah, Jesse Heitner, Mary Bernadette Kerubo, Kenneth Ngure, Leeya F. Pinder, Katherine K. Thomas, Nelly Rwamba Mugo, Sarah Gimbel

**Affiliations:** 1 Department of Child, Family, and Population Health Nursing, School of Nursing, University of Washington, Seattle, WA USA; 2 Kenya Medical Research Institute, Nairobi, Kenya; 3 Division of Infectious Diseases, Massachusetts General Hospital, Harvard Medical School, Boston, USA; 4 Department of Global Health, University of Washington, Seattle, USA; 5 School of Public Health, Jomo Kenyatta University of Agriculture and Technology, Nairobi, Kenya; 6 College of Medicine, University of Cincinnati, Cincinnati, OH USA

**Keywords:** Human papilloma virus, Cervical cancer, Thermal ablation, Single visit, Screen-and-treat approach, Kenya, RE-AIM, CFIR, ORIC, Implementation science

## Abstract

**Background:**

An important cervical cancer prevention strategy in low- and middle-income countries (LMICs) has been single-visit screen-and-treat (SV-SAT) approach, using visual inspection with acetic acid (VIA) and ablative treatment with cryotherapy to manage precancerous lesions. While SV-SAT with VIA and cryotherapy have established efficacy, its population level coverage and impact on reducing cervical cancer burden remains low. In Kenya, the estimated cervical cancer screening uptake among women aged 30–49 is 16% and up to 70% of screen-positive women do not receive treatment. Thermal ablation for treatment of precancerous lesions of the cervix is recommended by the World Health Organization and has the potential to overcome logistical challenges associated with cryotherapy and facilitate implementation of SV-SAT approach and increase treatment rates of screen-positive women. In this 5-year prospective, stepped-wedge randomized trial, we plan to implement and evaluate the SV-SAT approach using VIA and thermal ablation in ten reproductive health clinics in central Kenya.

**Methods:**

The study aims to develop and evaluate implementation strategies to inform the national scale-up of SV-SAT approach with VIA and thermal ablation through three aims: (1) develop locally tailored implementation strategies using multi-level participatory method with key stakeholders (patient, provider, system-level), (2) implement SV-SAT approach with VIA and thermal ablation and evaluate clinical and implementation outcomes, and (3) assess the budget impact of SV-SAT approach with VIA and thermal ablation compared to single-visit, screen-and-treat method using cryotherapy.

**Discussion:**

Our findings will inform national scale-up of the SV-SAT approach with VIA and thermal ablation. We anticipate that this intervention, along with tailored implementation strategies will enhance the adoption and sustainability of cervical cancer screening and treatment compared to the standard of care using cryotherapy.

**Trial registration:**

NCT05472311.

Contributions to the literature

- Application of an evidence-based, contextually appropriate, and replicable implementation strategy to improve screening and treatment of precancerous lesions of the cervix in low- and middle-income country settings.
- Rigorous evidence of impact on screening and treatment uptake using the single-visit and screen-and-treat approach with VIA and thermal ablation.
- Generate data on budget impact of the strategy to support scale up and sustainability planning.

## Background

Cervical cancer is almost entirely preventable with current technologies, yet it remains the fourth most common cause of cancer incidence and mortality among women globally [1]. Ninety percent of cervical cancer cases and deaths occur in low- and middle- income countries (LMICs) [2]. In response to this public health problem, the World Health Organization (WHO) issued a call in 2018 to eliminate cervical cancer globally [3]. In Kenya, cervical cancer is the second most prevalent cancer and the most common cause of cancer death in women, with an age standardized incidence of 33.8/100,000, resulting in 5236 cases and 3211 deaths in 2020 [4]. Despite the well-documented need for cervical cancer prevention, just 16% of Kenyan women (age 30–49) were screened in 2015 [5], far below the WHO’s target of 70% by 2030 [6]. For this reason, the Kenya National Cancer Control Strategy 2017–2022 prioritizes early detection strategies for cervical cancer [7].

The current WHO guideline recommends women over 30 years old be screened for cervical cancer every 3 years via visual inspection with acetic acid (VIA), Lugol’s iodine, or cytology when human papillomavirus (HPV) testing is not available as a primary screening modality [8]. The single-visit, screen-and-treat approach (SV-SAT) with VIA to detect and manage precancerous or cancerous lesions has been recommended for cervical cancer prevention in LMICs like Kenya because of its low cost and facilitation of treatment uptake among screen-positive women compared to cytology or HPV testing [9, 10]. VIA has demonstrated effectiveness in detecting cervical cancer at the population level in LMICs such as Zimbabwe [11–13]. However, implementation and maintenance cervical cancer screening programs in Kenya has been suboptimal due to multi-level barriers [14]. At the individual/patient level, low population-level awareness of screening services has impeded the uptake [15]. Other studies have also reported structural barriers such as attrition of trained personnel and supply and equipment stockouts [15, 16].

In the SV-SAT approach, women who screen positive for precancerous lesions are immediately treated with ablative therapy such as cryotherapy or thermal ablation, which minimizes loss to follow-up or attrition of screen-positive women [17, 18]. According to the 2015 Kenya STEPwise survey report on non-communicable diseases [5], up to 70% of screen-positive women do not receive treatment [19]. Low fidelity of the SV-SAT approach and treatment with cryotherapy in Kenya has been attributed to programmatic and logistical challenges of implementing cryotherapy in low-resource settings [15]. For example, large tanks of compressed refrigerant gas are difficult to supply and transport consistently to remote areas [14, 15, 20]. The equipment (cryotherapy probe and gas) is costly, and clinics that maintain cryotherapy equipment in Kenya have reported challenges with equipment failure and high turnover of trained personnel [21]. The 2019 WHO recommendations have added thermal ablation as another ablative treatment method for precancerous lesions [22]. The portable thermal ablation device can be charged with electricity, batteries, or solar panels, which is ideal for low-resource settings [23]. While the safety, efficacy, and acceptability of thermal ablation to women has been demonstrated in limited settings in parts of Zambia and Kenya [20, 24, 25], the drivers of its implementation and scale-up have not been determined.

Implementation strategies of evidence-based interventions (EBIs) such as SV-SAT with VIA and thermal ablation can facilitate identification of multi-level determinants of cervical cancer screening and treatment uptake and tailor EBIs to the local contexts to enhance their adoption and sustainment. We hypothesize that this intervention, along with tailored implementation strategies, will enhance the adoption and sustainability of cervical cancer screening and treatment compared to the standard of care using cryotherapy. Our specific aims are to:

- Aim 1) Develop locally tailored implementation strategies using multi-level participatory method with key stakeholders (patient, provider, system-level)
- Aim 2) Implement the intervention and evaluate clinical and implementation outcomes using the RE-AIM framework.
- Aim 3) Assess the budget impact of the intervention compared to single-visit, screen-and-treat method using cryotherapy.

We anticipate that the findings from this project will inform wider implementation and scale-up SV-SAT approach with VIA and thermal ablation and provide critical evidence to guide national policy and serve as a model for cervical cancer prevention efforts in similar LMIC settings. (NCT05472311).

## Methods

### Study design

**Fig. 1:**
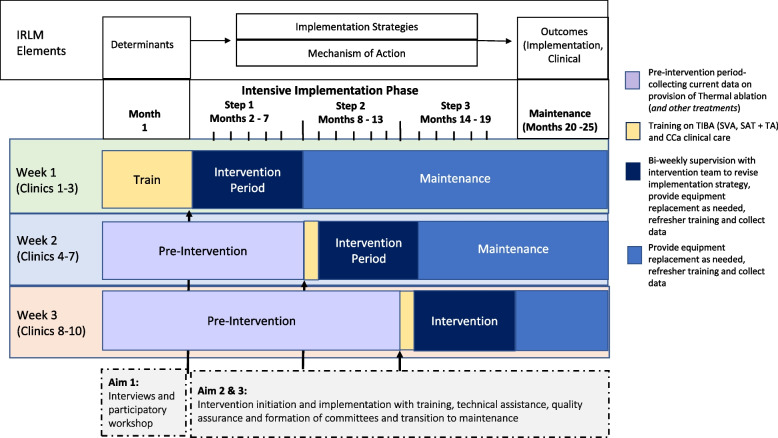
Conceptual model of the TIBA trial

This 5-year prospective, stepped-wedge, cluster randomized trial (hereafter referred to as “TIBA,” and translating to *cure/treatment* in Kiswahili, the Kenyan national language) aims to develop and evaluate implementation strategies to inform the national scale-up of SV-SAT approach with VIA and thermal ablation. We will initially use qualitative research with a participatory approach to develop an implementation strategy to introduce the intervention (SV-SAT with VIA and thermal ablation) in ten reproductive health clinics in central Kenya. Subsequently, we will evaluate the implementation of the intervention following the stepped-wedge cluster randomized trial design. The conceptual framework for this trial was informed by the implementation research logic model (IRLM, Fig. 1). The IRLM has been described extensively in the literature, but briefly, involves identifying and specifying the relationship between the determinants, implementation strategies, mechanism of action, and outcomes of evidence-based interventions [26]. A mixed-methods evaluation will assess implementation success. The Reach, Effectiveness, Adoption, Implementation and Maintenance (RE-AIM) framework will be used to assess the impact, which has demonstrated capacity to guide assessments of the public health impacts of complex intervention by capturing formative, process and outcome dimensions on the individual, organizational, and policy levels [27]. We will also use the Consolidated Framework for Implementation Research (CFIR) to guide the data collection and interpretation of the implementation outcomes and determine necessary adaptations in delivery as well as organizational determinants of successful implementation [28]. The CFIR is a meta-framework that incorporates components from evidence-based implementation process theories, guides intervention planning and implementation, and identifies contextual influences that explain the heterogeneity of implementation success across settings. This meta-framework is organized into five domains, comprised of 39 constructs and serves as a guide to identify core and adaptable components, an essential requirement to support scaling of interventions and has been applied successfully in LMICs settings [29]. The use of these established implementation science frameworks will help ensure our evaluation provides actionable, appropriate, and holistic implementation guidance to scale SV-SAT with VIA and thermal ablation across heterogeneous health facilities. As part of this comprehensive evaluation, we will also assess the cost and budget impact of the intervention, which is critical for policymakers in LMICs to define priorities and actualize realistic and effective planning. Our project will produce a comprehensive delivery guide in collaboration with the Kenya National Cancer Control Program (NCCP) along with implementation guidelines for scaling nationwide. The CONSORT extension for stepped-wedge randomized trials and the Standards for Reporting Implementation Studies (StaRI) Statement are available in additional materials [30].

### Setting

We will implement this work in ten health facilities in Embu, Kiambu, Nairobi, and Murang’a counties in central Kenya, including urban faith-based, rural, and urban health facilities. The sample is purposively selected to be nationally representative and heterogeneous in order to provide contextually relevant guidance applicable for national scaling of the intervention. The eligibility to participate will include the following: (1) Hospital levels 3–5 according to the Kenyan level system, public or private medical facilities; (2) current use of VIA as the primary screening method; and (3) cervical cancer screening volume over a 4-month period (greater than 1000) [31]. Additional factors such as geographical setting (urban vs. rural), leadership/management structure, funding mechanism (public vs. private/charity), number of providers trained in cervical cancer screening and their scope of practice, and patient fee structure for screening as well as screening volume will be considered. Site level variation is critical to scale the intervention in heterogeneous clinics with different management and staffing systems.

### Randomization

Three to four clinics will be randomly assigned to one of three 6-month implementation waves at the stakeholder meeting at the conclusion of year 1. The clinics will be stratified by size.

### Aim 1: develop locally tailored implementation strategies using multi-level participatory method with key stakeholders (patient, provider, system-level)

The intervention will include cervical cancer screening and treatment using SV-SAT with VIA and thermal ablation. To build on existing data from the region and the investigative team, we will use participatory methods to conduct qualitative interviews with clients, front-line health providers, facility managers, and policy makers in the Ministry of Health to assess and address barriers and facilitators to uptake and provide information on the introduction of the intervention through stakeholder engagement. Based on informal engagement with frontline health care workers and managers in the study area, we anticipate openness to the intervention. Existing barriers will be identified early in project implementation to address any unforeseen challenges to implementation. At the end of year 1, we will hold a stakeholder workshop to map out context-specific strategies that address potential barriers to intervention uptake and account for heterogeneity in provider and system inputs across facilities. Information gathered during these research activities will help investigators refine the essential components of the implementation strategy to be evaluated in Aim 2.

#### In-depth qualitative interviews

In-depth interviews will use semi-structured topic guides, to explore barriers and facilitators to potential adoption, delivery, and sustainment of the intervention. We will also interview frontline health providers carrying out cervical cancer screening in reproductive health clinics (*n* = 20). These health providers will be purposively sampled by profession to ensure representation of nurses, clinical officers, and doctors who provide reproductive health care, as well as a subset with management roles (*n* = 10), and two female patients recently screened for cervical cancer in each potential study facility (*n* = 20). Sample size determination in qualitative research rests on maximizing potential for saturation (when new interviews do not meaningfully add to codes and themes already represented in the previously collected data). Based on experience, we fully envision reaching saturation with (*n* = 5) policymakers stationed at the National Cancer Institute and the Division of Reproductive Health at the Ministry of Health of Kenya. Our investigative team has extensive experience conducting in-depth interviews with health providers.

#### Qualitative data analysis

Digital recordings of the interviews will be transcribed. We will then use a deductive framework method with a combination of deductive and inductive analysis to allow for exploration of specific themes but also allow space to discover other unexpected aspects of participants’ experiences. We will use the qualitative data management computer program Dedoose, which will then be repeatedly sorted and re-reviewed by two experienced social scientists to identify a broader set of concepts [32].

#### Stakeholder workshop

To complement these interviews, we will conduct a 1-day stakeholder workshop, in collaboration with the National Cancer Institute to present findings from the qualitative work and introduce our study, specifically the phased introduction of intervention and the proposed implementation strategy development and refinement process. We anticipate the meeting size will be approximately 40–50 people. Invitees will include representation from the National Cancer Institute, Ministry of Health leadership, county-level reproductive health coordinators, and health facility managers and select front line health providers from participating reproductive health facilities that provide VIA or are interested in doing so. At this workshop, we will engage stakeholders in mapping exercises to identify how services for cervical cancer screening and treatment are currently provided across each site. The results of the mapping and the qualitative findings will be reviewed to identify categories of determinants driving implementation at the facility level. Baseline conceptual framing of the implementation process will be introduced using IRLM so facility level teams can conceptually link determinants, their implementation strategies, and the hypothesized mechanisms of action to implementation and clinical outcomes. Throughout the stakeholder meeting process, we will invite stakeholders to suggest preconditions and/or adaptations to the planned implementation of the intervention that may be necessary in specific local contexts and settings, and we will attempt to incorporate ways to include these implementation guidelines for the sustainable introduction of the intervention. Specifically, we will use this feedback to refine our design to be compatible with facility workflow and systems, aligns with organizational culture, and is agreeable to our stakeholders. Finally, at this workshop, we will collectively randomize clinics into one of three phases to begin implementation. The randomization will be performed in a public manner in order to ensure organizational receptivity and to build ownership of the implementation process.

#### Data collection and abstraction

The data on facility-level infrastructure including availability of equipment, supplies, and current staffing (numbers, cadre and training) and patient flow will be obtained. Infrastructure supplies and staffing data collection will be collected at three time points in the ten RH study facilities; before and after each implementation wave and at the end of the maintenance phase. Routine data on the proportion of women screened, screened positive, and treated for abnormal lesions will be abstracted monthly, by research staff, covering a minimum period of 6 months prior to introduction of the intervention at any facilities and continuing through the end of the maintenance phase. During pre-implementation periods, clinics will contribute data to the “control” condition in the stepped wedged trial.

### Aim 2: implement the intervention and evaluate clinical and implementation outcomes using the RE-AIM framework

#### Overview

At 10 reproductive health clinics in central Kenya, we will introduce the intervention and site-specific implementation strategy developed in Aim 1 in a stepped-wedge fashion and rigorously evaluate how effectively this strategy is disseminated and implemented. In three successive 6-month waves, we will roll out the intervention at three to four clinics. A maintenance phase of varying lengths will be conducted across all waves.

#### Power and sample size

The stepped-wedge study design is powered based on the primary effectiveness outcome, which is the proportion of screen-positive women who receive same-day treatment. According to data on cervical cancer screening and preventive treatment for Kiambu county from 2017 to 2019, 20% of cervical cancer screening clients who screened positive received cryotherapy or LEEP. Routine data from our proposed study facilities show an average of 30 clients per month screened at each facility in the first 3 months of 2020. Assuming 6-month waves, 30 clients screened per month and a 5% screen positive rate, we expect an average of 9 screen-positive women per facility per wave. Assuming an intracluster correlation coefficient of 0.2, alpha = 0.05, and 20% treatment coverage pre-intervention, we will have 80% power to detect a three-fold increase to 60% treatment coverage with the intervention (R [v 3.6.3] package swCRTDesign [v 3.1]). We estimate that 90% of screen-positive women will be eligible for ablative treatment.

#### Training

Before the implementation of the intervention, we will train health providers in participating reproductive health clinics, with a refresher course on VIA and thermal ablation treatment techniques as part of single visit as per IARC guidelines. The training curriculum will be adapted to address gaps identified in year 1. On-site hands-on training will be done at each facility with support of research training team for a duration of 4 days. Outreach community activities will be done prior to scheduled on-site training to provide an adequate number of clients for screening and demonstration of thermal ablation. Each trained provider will be required to complete a set number of procedures before they receive a proficiency certificate. The selected reproductive health clinics have some of their providers trained on VIA and cryotherapy. Our team of investigators has three reproductive health experts with thermal ablation training skills who will develop and train a team of trainer of trainers at each reproductive health clinic. Regular training at each clinic will be provided with engagement of a Kenyan consultant gynecologist to provide consultant training services for the trainer of trainers for the initial 2 clinics. Thereafter, the research team and the county trainer of trainers will provide on-site hands-on training. Training will be led by specialists skilled in colposcopy, loop electrosurgical excision procedure (LEEP), cryotherapy, and thermal ablation. Quarterly CME and refresher training during implementation will be done. In addition to thermal ablation training, CME-type training on cervical cancer prevention interventions and refresher didactic training on VIA will be performed.

#### Provision of technical assistance

We will provide bi-monthly technical support from the study team with assessment of fidelity and tailoring of the implementation strategy for 6 months at all sites. Depending on their wave assignment, sites will move to a maintenance phase of 18 (wave 1), 12 (wave 2), or 6 (wave 3) months of length as described in Fig. 1. Services provided during the intervention phase include bi-monthly supervision, data collection support, equipment and supplies related to the intervention, and ongoing clinical training for new staff. After the intervention phase bi-monthly, supervision is no longer provided but we will offer intermittent training where necessary and continue data collection. In Aim 2, reproductive health clinics will implement the intervention with the implementation strategy developed and refined at the year 1 stakeholder meeting. We expect that implementation strategies across the 10 sites will include some common and some site-specific components. During the 6-month intervention period, facilities will be supported by the study team to iteratively tailor their implementation strategy using their IRLM conceptual map and identified and prioritized strategy components. Any subsequent adaptations to the site-specific implementation strategies during the intervention period will be documented through use of the FRAME-IS tool [33] including collation of what is modified, the timing of the modification, and the actors and action targets of the modification.

#### Formation of facility committees

We will convene a committee that will serve an advisory team at the facility level. This committee will work closely with the research team to provide input on on-project implementation processes and receive updates provided by the research team. The research team will liaise with the committees to schedule regularly scheduled meetings. We will evaluate the impact of the intervention on the clinical and implementation outcomes using the RE-AIM framework (Table 1).

**Table 1:** Summary of outcomes, by RE-AIM domain

RE-AIM domain	Outcomes	Data source
**Reach**	• Proportion of providers trained and women screened per month	• Abstracted client records • Facility level data
**Effectiveness**	• Number of women screen-positive per month • Treatment completion rate among screen-positive women compared to pre-intervention (primary) • Proportion of women with clearance of HPV 6 months post-receiving TA treatment	• Facility level data • HPV test results from randomly selected women
**Adoption**	• Proportion of selected clinics incorporating thermal ablation into routine care through updated policies • Proportion of trained providers performing SV-SAT + TA • Facility readiness	• Facility level data, Technical assistance reports • ORIC scores
**Implementation**	• Core components of the SV-SAT + TA • Description of drivers of success/failure using CFIR constructs of interest	• Facility level data, Technical assistance reports • Key informant interviews
**Maintenance**	• Proportion of clinics that continue to provide and sustain SV-SAT + TA services 12,18, and 24 months after intervention implementation	• Facility level data

### REACH

For reach of the intervention, we will assess the number of providers trained within each clinic. We hypothesize that the intervention will increase the number of women receiving cervical cancer screening and treatment services in all facilities. We will abstract program data and estimate the number of women aged 25–49 years (according to the Kenyan national screening guidelines) seeking services in reproductive health clinics who are screened per month [34].

### Effectiveness

We will assess effectiveness by determining whether the intervention is associated with an increase in the number of screen-positive women identified per month and proportion of screen-positive women who complete treatment (target = 60%) comparing pre-implementation period to intervention periods. This will include women treated with thermal ablation, cryotherapy, LEEP, or any other procedure.

#### Data analysis

The primary analysis will be an individual level analysis comparing the primary efficacy outcome (probability of screen-positive women being treated the same day) pre- vs post-intervention. We will estimate differences using weighted least square methods appropriate for this facility-randomized three-wave stepped-wedge design, where all clinics begin in the control condition. We will consider the intervention effect to be fixed throughout the post-intervention period, but random intercepts per facility and wave. Within the post-intervention period, we will examine whether the effect waned in the maintenance phase using an analysis comparing the intensive vs maintenance phases. To analyze secondary outcomes of probability of providers being trained and number of women screened (REACH outcomes) and number screen-positive (secondary efficacy outcome), we will use the same analysis approach. Exploratory analysis will examine potential effect modification of facility-level factors such as patient volume, patient to provider ratio, and women screened. Potential multi-level adjustment variables include individual-level factors such as age.

#### Adoption

To assess adoption, we will assess (1) facility readiness to implement the intervention, (2) the proportion of clinics incorporating thermal ablation into routine care through updated policies, and (3) proportion of trained providers performing the single-visit screen-and-treat approach to the screen-eligible women and thermal ablation for screen positive women.

To assess readiness for intervention adoption, we will apply the validated ORIC assessment scale translated into Kiswahili and adapted to the implementation context after each wave begins. ORIC is a 12-item Likert-type scale, broken into domains of change commitment (4 items) and change efficacy (8 items), and has demonstrated reliability, content validity, structural validity, structural invariance, and known-groups validity in field application. We will apply the ORIC to 3 management team members of each intervention facility (*n* = 30) and 6 frontline health workers per intervention facility (*n* = 60). Analysis will test whether sufficient inter-rater reliability and inter-rater agreement exist to aggregate individual responses to the facility level. If tests do not justify aggregation, we will use a measure of intra-facility variability in readiness rather than a facility-level mean in our analysis. The resulting analysis will provide readiness profiles for each facility as they initiate implementation, which will complement adoption, implementation, and effectiveness data in understanding the broader impact of the intervention.

Information on the proportion of clinics incorporating thermal ablation into routine care and the proportion of trained providers performing the SV-SAT approach to the screen-eligible women and thermal ablation for screen positive women will be collected every 2 weeks by the technical assistants during the monitoring visits.

### Implementation

Implementation will be measured at the organizational levels to establish consensus on the core components of the intervention as well as determine the drivers of its implementation success and failure. CFIR will guide our examination of the implementation processes and adaptations across the different facilities as well as document determinants of success and failure. We will conduct small group discussion with health providers (*n* = 3) at each clinic and in-depth interviews with health managers (*n* = 10) and screen-positive women (2 per clinic, *n* = 20) and spread out over the intensive implementation wave. Interviewees will be purposively selected to capture diversity in health facilities (rural vs urban, large vs small size, government facility vs mission facility). Interview guides will include questions adapted from the CFIR question bank (cfirguide.org) to gather data about selected constructs from 4 of the established CFIR domains (Table 2). Outer setting constructs of interest will be captured via fields to identify critical external events. Questions associated with these novel constructs will be piloted for understandability and appropriateness prior to data collection. Constructs of interest may be adapted based on the outcomes of Aim 1.

**Table 2:** Potential questions to providers and managers by CFIR construct

Intervention characteristics	
• Relative advantage • Adaptability • Complexity • Perceived scalability	• How does thermal ablation compare to cryotherapy in your setting? • ﻿﻿What kinds of changes or alterations do you think you will need to make to SV-SAT + TA so it will be effective? • ﻿﻿How complicated is use of thermal ablation? • ﻿﻿Do you think TA could be expanded for use in other sites?
Outer setting	
• Patient needs and resources	• How well do you think SVA-SAT + TA will meet the needs of women screening for cervical cancer in your facility? • Have you heard stories about the experiences of participants with the SV-SAT + TA?
Inner setting	
• Structural • Characteristics • Networks • Tension for change • Compatibility • Team characteristics • Collective efficacy	• How will the infrastructure of your facility affect the implementation of SV-SAT + TA? • What kinds of infrastructure changes will be needed to accommodate SV-SAT + TA? • Are meetings such as staff meetings, held regularly? What is discussed? Is there a strong need for SV-SAT + TA? Why or why not? • How well does SV-SAT + TA fit with existing work processes and practices in your setting? What are likely issues or complications that may arise? • What sort of team-based care is provided in the facility? • Is your healthcare team capable of working together to implement new interventions like SV-SAT + TA?
Characteristics of individuals	
• Self-efficacy	• How confident are you that you will be able to successfully implement SV-SAT + TA? • What gives you that level of confidence?
Process	
• Executing • ﻿﻿Champions • ﻿﻿Decision-making	• Has SV-SAT + TA been implemented according to the implementation plan? [If Yes] Can you describe this? [If No] Why not? • Are there people in the facility who are likely to champion the intervention? • How are decisions made in this organization? Who make decisions in this organization?
Characteristics of systems	
• Systems architecture • External funding Agent • Priorities • Strategic policy Alignment • Resource continuity	• How is the health system organized and how does it interact with other systems that contribute to the health of the public and that influence how the CC programs are designed and implemented? • ﻿What are the priorities and perspectives of external donors related to TA and CC in general in-country? • ﻿What policies exist in country that support integration of TA? • What existing or potential funding sources exist to continue and scale TA?

### Fidelity to the intervention (HPV sub-study)

We will assess fidelity to thermal ablation training by assessing for HPV clearance among HPV-positive women. We will collect HPV cervical swab prior to and 6 months post-treatment to assess for clearance of type-specific HPV infection (target 60% HPV clearance) post-treatment on all screen positive women treated with thermal ablation. HPV samples will be shipped to the UW/UON HIV/STD Research laboratory in Mombasa. Assuming at least 200 women consent to the HPV sub-study and 70% of them test positive for HPV prior to thermal ablation (*N* = 140), the largest confidence interval for HPV clearance will be ± 8.1%.

### Maintenance

In the first 6 months of implementation in each wave, we will have bi-monthly on-going technical support to health facilities. Thereafter, we will reduce visits to once a month among clinics that meet metrics for transition, and we will then discontinue routine technical assistance visits, but only respond to facility requests as needed. Over this time, research staff will continue to abstract individual level programmatic data. We will continue to monitor the proportion of screen-positive women who complete treatment. In addition, we will monitor the frequency of facility committee meetings. We anticipate that facilities will continue to implement the intervention because we will have utilized a participatory approach that builds ownership and organizational commitment and results in implementation of an intervention that is contextually fit.

### Aim 3. Assess the budget impact of the intervention compared to single-visit, screen-and-treat method using cryotherapy

We will quantify and compare the costs of the intervention to the costs of the current recommended standard practice of SV-SAT with cryotherapy. Using micro-costing techniques, we will estimate the programmatic costs of each strategy, including implementation costs. We will use these data to estimate the cost per woman treated under each strategy, and we will incorporate reach and effectiveness outcome data (from Aim 2) in a decision analysis model to estimate and compare the costs incurred and averted. Cost and impact data will be used to estimate the budget impact of SV-SAT with each treatment strategy. Our primary analyses will take the programmatic perspective, and secondary analyses will include costs incurred by patients to access services and duration of time used by providers to provide services. Our approach, analyses, and results reporting will align with published economic evaluation guidelines, which will maximize the transparency and generalizability of our findings to other settings [31, 32]. We hypothesize that SV-SAT with VIA and thermal ablation will cost less per woman screened and treated and will increase the affordability, feasibility, and impact of cervical cancer prevention relative to SV-SAT with cryotherapy.

### Micro-costing procedure

To capture heterogeneity in costs and efficiency across settings, we will collect cost data in a representative sample of six reproductive health clinics. We will use an activity-based micro-costing approach to measure start-up, implementation, personnel, supply, capital, overhead, and patient costs in each clinic with standard practice (SV-SAT with cryotherapy) and with implementation of the intervention (Table 3). Measured costs will include those incurred in the initial screening and treatment visit as well as follow-up visits. Following published protocols, we will observe clinic visits using time-driven activity-based costing (TDABC) methods to map out and quantify the time spent on each component activity [33]. We will also conduct semi-structured interviews with staff and key personnel to inform estimates of the time and costs required for provision of relevant SV-SAT services. TDABC and interview data will be used to estimate the personnel time required for different cadres of personnel, which will be translated into costs based on average salaries. Start-up costs, ongoing implementation costs, and other program costs will be obtained from project expense reports, clinic records, Ministry of Health (MOH) records, and published literature, as in previous studies [21, 34,35]. Patients will be asked about out-of-pocket expenses and time losses, with time losses translated into costs using data on local wages.

**Table 3:** Summary of cost measures and associated data sources

Cost type	Elements	Data sources
Start-up	Training materials: training expenses (trainer fees, staff time spent in training)	Project expense reports; project staff/civil service salaries
Implementation	Ongoing training expenses; technical assistance support, facility committee meetings, fidelity monitoring; community outreach	Project expense reports
Personnel	Time/salaries of healthcare personnel, laboratory personnel, supervisors, and administrative staff	TDABC observations, staff interviews, civil service salaries
Patient	Transportation expenses; other out-of-pocket expenses, including childcare; time losses	Patient surveys, local average wages
Supplies	Consumables such as acetic acid, CO_2_ or N_2_O refrigerant gas, specula, anesthesia, equipment maintenance; transportation costs	Clinic and project expense reports; MoH records
Capital	Liger thermal ablator; cryotherapy machine; laboratory equipment; vehicles	Clinic and project expense reports; MoH records
Overhead	Utilities; clinic maintenance	Study clinic records and expense reports; staff interviews

We will summarize data on costs to estimate the total cost of the intervention per woman. Our micro-costing approach will allow us to identify and exclude costs specific to research activities, focusing only on programmatic costs. To reflect variable costs and activities in different phases of implementation of the intervention, we will stratify costs by initial start-up, implementation and maintenance phases. For each strategy, we will break costs down into the categories in Table 3, above, to identify the key cost drivers. Costs will be discounted at 3% per year, and we will explore alternative discounting rates of 5% and 0%.

### Budget impact analysis procedure

To account for differences in reach and effectiveness by treatment option, we will develop a decision tree model to estimate the expected number of CIN2 + and cervical cancer cases that would arise with SV-SAT with thermal ablation relative to SV-SAT with cryotherapy given the size of the eligible patient population in Kenya. To estimate these health outcomes, we will incorporate study data on reach and effectiveness with each strategy (Aim 2) and published data on HPV natural history and progression to cancer. Costs will be estimated using study data on the cost per woman screened and treated with the intervention and published data of the costs of cervical cancer screening and treatment. To account for variability and uncertainty in key parameters, we will conduct sensitivity analyses by jointly varying these parameters within observed or probable ranges. The model will be programmed using TreeAge Software (Williamstown, MA) [35]. Using data on current Kenya MOH expenditures, we will estimate the budget impact of the two interventions from the MOH perspective. This analysis will reflect the opportunity costs incurred by delivery of the intervention as well as costs averted through prevention of cervical cancer cases.

## Discussion

The intervention of SV-SAT with VIA and thermal ablation can be a crucial component in reaching cervical cancer elimination in LMICs. Our study proposes to develop and evaluate implementation strategies to inform the national scale-up of the intervention. Findings from our study will inform scale-up of SV-SAT with VIA and thermal ablation to both national and county governing bodies and support decision-making regarding allocation of resources towards training of health care providers and providing equipment.

## Data Availability

Data sharing is not applicable to this article as no datasets were generated or analyzed during the current study.

## Abbreviations

CC: Cervical cancer
SVA: Single-visit approach
SAT: Screen and treat
LMICs: Low- and middle-income countries
WHO: World Health Organization
TA: Thermal ablation
D&I: Dissemination and implementation
RE-AIM: Reach, Effectiveness, Adoption, Implementation and Maintenance
RH: Reproductive health
IDI: In-depth interviews
CFIR: Consolidated Framework for Implementation Research
HPV: Human papilloma virus
DHIS: Demographic Health Information System
LEEP: Loop electrosurgical excision procedure
VIA: Visual inspection with acetic acid
VILI: Visual inspection with Lugol’s iodine
IRB: Institutional review board
KEMRI: Kenya Medical Research Institute
CIN: Cervical intraepithelial neoplasia
NIH: National Institutes of Health (US)
HIV: Human immunodeficiency virus
ORIC: Organizational readiness for implementing change
STI: Sexually transmitted infection
PrEP: Pre-exposure prophylaxis
IS: Implementation science
NCCP: National Cancer Control Program
KMET: Kisumu Medical and Educational Trust
US: United States
UW: University of Washington
VCT: Voluntary counseling and testing
MoH: Ministry of Health
PHRD: Partners in Health and Research Development
ICRC: International Clinical Research Center
CME: Continuous medical education
QA: Quality assurance
UoN: University of Nairobi
TDABC: Time-driven activity-based costing
